# Report of Section II

**Published:** 1895-12

**Authors:** Louis Ottofy

**Affiliations:** Chicago


### Report of Section II.

BY LOUIS OTTOFY, D.D.S., CHICAGO.

Abstract of report presented at the American Dental Association, Aug., 1895.

At the close of the session of 1894 the total number of dental colleges in active operation, and granting degrees was forty-seven. There has been established since that time one dental college, the Dental Department of the University of Omaha, making a total of forty-eight now in active operation, and granting degrees.

Dr. Ottofy then gave statistics regarding the number of students in the schools during the year past. There were matriculated as students of dentistry during the past year 5,277 persons, upon 1,226 of whom the dental degree was conferred. Four years ago, twelve of the thirty-three colleges then existing graduated three-fourths of the entire number. Three years ago, ten colleges graduated 851 students, while the remaining twenty-eight graduated 632. This year six colleges graduated 525, the remaining forty-one, 701.

REPORT ON DENTAL LITERATURE.

The most important work of the year, and the most valuable additions to dental literature in its history, is the Transactions of the World's Columbian Dental Congress, which appeared in December last, in two volumes. There has been a movement for some time to introduce the term "stomatologist" in place of the word "dentist," and during the past year one periodical dental journal has taken this step.

EQUAL parts of glycerine and castor oil, to which is added a little oil of cinnamon, makes a pleasant purgative for children.

